# Convergência de políticas públicas educacionais na promoção da alimentação adequada e saudável

**DOI:** 10.26633/RPSP.2019.96

**Published:** 2019-11-27

**Authors:** Solange Fernandes de Freitas Castro, Mariana Belloni Melgaço, Vanessa Manfre Garcia de Souza, Karine Silva dos Santos

**Affiliations:** 1 Ministério da Educação Fundo Nacional de Desenvolvimento da Educação Brasília (DF) Brasil Ministério da Educação, Fundo Nacional de Desenvolvimento da Educação, Brasília (DF), Brasil.

**Keywords:** Educação alimentar e nutricional, alimentação escolar, programas e políticas de nutrição e alimentação, segurança alimentar e nutricional, Brasil, Food and nutrition education, school feeding, nutrition programs and policies, food and nutrition security, Brazil, Educación alimentaria y nutricional, alimentación escolar, programas y políticas de nutrición y alimentación, seguridad alimentaria y nutricional, Brasil

## Abstract

O presente relato tem como objetivo descrever a trajetória de convergência do Programa Nacional de Alimentação Escolar (PNAE) com o Programa Nacional do Livro e do Material Didático (PNLD) no Brasil, com destaque para o projeto de inclusão dos temas de alimentação e nutrição nas capas de livros didáticos distribuídos de forma universal e gratuita a todas as escolas públicas brasileiras. Essa iniciativa intersetorial dialoga diretamente com as recomendações do Guia Alimentar para a População Brasileira, com os princípios do Marco de Educação Alimentar e Nutricional para as Políticas Públicas e com as propostas da Década da Ação em Nutrição das Nações Unidas. Verificou-se que a construção de um espaço dialógico e intersetorial é um caminho possível para a promoção da alimentação adequada e saudável aos estudantes. Entretanto, sugere-se a realização de estudos que avaliem a efetividade da inserção dos temas de alimentação e nutrição nos livros didáticos e que investiguem a percepção dos docentes e discentes quanto ao uso dos livros para promover a educação alimentar e nutricional.

## Abstract (en)

The present report describes the process of convergence between the Brazilian National School Feeding Program (PNAE) and the Brazilian National Textbook Program (PNLD), highlighting a project that introduced diet and nutrition topics on the covers of textbooks distributed at no cost to all public schools in Brazil. This intersectoral initiative speaks directly to the recommendations of the Dietary Guidelines for the Brazilian Population, the principles of the Nutrition Education Framework for Public Policies, and the proposals laid out in the United Nations Decade of Action on Nutrition. The report shows that the construction of a dialogic and intersectoral space is a possible path to promote a healthy and appropriate diet among students. However, further studies are necessary to evaluate the effectiveness of introducing diet and nutrition topics in textbooks, and also studies investigating the perception of teachers and students regarding the use of books to promote education on diet and nutrition.

## RESUMEN

El objetivo del presente informe es describir la trayectoria de convergencia del Programa Nacional de Alimentación Escolar (PNAE) con el Programa Nacional de Libros y Materiales Didácticos (PNLD) en Brasil, con hincapié en el proyecto de inclusión de los temas de alimentación y nutrición en las portadas de los libros didácticos distribuidos de manera general y gratuita a todas las escuelas públicas brasileñas. Esa iniciativa intersectorial guarda una relación directa con las recomendaciones de la guía alimentaria para la población brasileña, los principios del marco de educación alimentaria y nutricional para las políticas públicas y las propuestas del Decenio de las Naciones Unidas de Acción sobre la Nutrición. Se comprobó que la construcción de un espacio dialógico e intersectorial es una posible vía para fomentar el consumo de una alimentación adecuada y saludable en los estudiantes. Sin embargo, se recomienda realizar estudios para evaluar la efectividad de la inclusión de los temas de alimentación y nutrición en los libros didácticos e investigar la forma en que el cuerpo docente y discente percibe el empleo de los libros para promover la educación alimentaria y nutricional.

No Brasil, a Constituição Federal de 1988 institucionalizou a educação e a alimentação como direitos fundamentais. No que se refere ao dever do Estado com a educação, incluídas as três esferas governamentais (União, estados e municípios), declarou que o direito à educação deverá ser efetivado mediante a garantia de atendimento gratuito e universal a estudantes de 4 a 17 anos de todas as etapas da educação básica, por meio de programas suplementares de material didático escolar, transporte, assistência à saúde e alimentação (1).

Esses princípios constitucionais estão em consonância com as propostas da Década da Ação em Nutrição das Nações Unidas (2016 a 2025) (2) proclamada pela Assembleia Geral das Nações Unidas (ONU) em 2016. A Década tem o potencial de propagar uma abordagem ampliada e articulada de enfrentamento de todas as formas de má nutrição, induzindo mudanças em políticas nacionais em diversos setores.

O Brasil, um dos primeiros países a se comprometer internacionalmente com a agenda da Década, entende que a má nutrição, em todas as suas formas (desnutrição, carências de micronutrientes, sobrepeso e obesidade), afeta não somente a saúde e o bem-estar das pessoas, mas também gera consequências sociais e econômicas relevantes (3). Sendo assim, o país assumiu, frente ao desafio da Década, compromissos alinhados às metas que já haviam sido estabelecidas no II Plano Nacional de Segurança Alimentar e Nutricional (PLANSAN), construído a partir da Política Nacional de Segurança Alimentar e Nutricional (PNSAN) (4, 5).

Dentre os seis pilares da Década, é no Pilar 3 – Proteção social e educação nutricional – que se enquadra o Programa Nacional de Alimentação Escolar (PNAE), instrumento estratégico de promoção da alimentação adequada e saudável e da garantia da segurança alimentar e nutricional. Dentre os programas suplementares à educação, o PNAE é gerenciado, juntamente com o Programa Nacional do Livro e Material Didático (PNLD), pelo Fundo Nacional de Desenvolvimento da Educação (FNDE), autarquia vinculada ao Ministério da Educação (MEC), responsável pela transferência dos recursos financeiros, apoio técnico, coordenação e estabelecimento das normas gerais de planejamento, execução, controle, avaliação, monitoramento e prestação de contas dos programas. Só em 2017 foram investidos mais de 5,5 bilhões de reais na execução dos programas educacionais de alimentação escolar e livro didático, atendendo a todos os estudantes da rede pública brasileira (6, 7).

Nesse contexto, o presente relato tem como objetivo descrever a trajetória de convergência do PNLD com o PNAE no Brasil, com destaque para o projeto de inclusão dos temas de alimentação e nutrição nas capas de livros didáticos distribuídos de forma universal e gratuita a todas as escolas públicas brasileiras.

## O PROGRAMA NACIONAL DE ALIMENTAÇÃO ESCOLAR

O PNAE se destaca como a política pública educacional de maior longevidade do país na área de segurança alimentar e nutricional. Ao longo dos seus 60 anos de história, o PNAE resistiu a diferentes orientações governamentais e, com o passar do tempo, foi aperfeiçoado a ponto de se transformar em uma das maiores políticas de segurança alimentar e nutricional do mundo (8).

Da criação do PNAE até 1993, a aquisição de gêneros alimentícios para a alimentação escolar era realizada de forma centralizada e distribuída para os municípios brasileiros. Entretanto, a partir de 1994, a alimentação escolar no Brasil passou a ser realizada de forma descentralizada, mas com a necessidade de celebração de convênio entre a União e os municípios, as secretarias estaduais de educação e o Distrito Federal (DF). Atualmente, os recursos financeiros para a execução do PNAE são consignados no orçamento da União e transferidos de forma voluntária pelo FNDE, sem necessidade de ajustes ou convênios. O FNDE repassa um valor *per capita* para os estudantes matriculados em todas as escolas de educação básica da rede pública. O cálculo desse valor tem como base o quantitativo de estudantes matriculados declarados pelos entes federados no censo escolar anual, realizado pelo Instituto Nacional de Estudos e Pesquisas Educacionais Anísio Teixeira (INEP).

Em 2009, a lei 11 947 (9) institucionalizou vários avanços anteriores do PNAE. Além disso, o Programa inovou ao universalizar o atendimento a todos os estudantes matriculados na rede pública de educação básica, contemplando escolas federais, filantrópicas, comunitárias e confessionais conveniadas com o poder público, inclusive as de educação especial.

Ainda outra inovação instituída pela lei 11 947/2009 foi a obrigatoriedade da aquisição de alimentos da agricultura familiar para a alimentação escolar. Essa é uma das características que consolidam o PNAE como política estruturante de segurança alimentar e nutricional, crucial para a efetivação do direito dos estudantes à alimentação adequada e saudável e para a melhoria das condições de vida dos agricultores e do desenvolvimento local (10-14). Ademais, a aquisição de alimentos da agricultura familiar se insere no contexto das ações de educação alimentar e nutricional (EAN) na medida que promove o fornecimento de alimentação adequada e saudável na escola, favorece hábitos alimentares regionais e culturais saudáveis (15) e permite o desenvolvimento de atividades de educação pautadas na produção e no consumo sustentável dos alimentos, assim como a utilização de uma abordagem integral ao sistema alimentar (16, 17).

É importante retratar o contexto de criação do marco legal do PNAE e da vinculação da agricultura familiar à alimentação escolar. Em 2006, a Lei Orgânica de Segurança Alimentar e Nutricional (LOSAN) criou o Sistema Nacional de Segurança Alimentar e Nutricional (SISAN) com o propósito de assegurar o direito humano à alimentação adequada (DHAA) (18); em 2010, o decreto 7 272 regulamentou essa lei e instituiu a PNSAN (19).

Segundo Custódio et al. (20), o PNAE se insere em dois eixos articuladores da PNSAN: o de acesso a alimentos e o de fortalecimento da agricultura familiar (20). Estudos mostram que a compra de alimentos da agricultura familiar para o PNAE pode promover melhorias na qualidade do cardápio escolar, devido, por exemplo, à maior diversidade de alimentos frescos, e contribuir para o desenvolvimento de hábitos alimentares saudáveis, configurando-se como importante ferramenta de EAN (16, 21, 22).

Além disso, todas as diretrizes do PNAE dialogam com as diretrizes da PNSAN, cabendo destacar a que diz respeito à instituição de ações de EAN (9, 19). Esse tema tem sido trabalhado por diferentes ministérios: da Saúde, da Educação e do Desenvolvimento Social e Combate à Fome (reestruturado no ano de 2018, sendo atualmente denominado de Ministério da Cidadania) – tendo este último lançado o Marco de Referência de Educação Alimentar e Nutricional para as Políticas Públicas (23, 24).

A lei 11 947/2009 (9) trouxe o conceito de alimentação escolar e delegou ao MEC, por meio do FNDE, a competência de propor ações educativas que perpassem o currículo escolar. Essas ações devem abordar o tema da alimentação e nutrição e o desenvolvimento de práticas saudáveis de vida na perspectiva da segurança alimentar e nutricional, fazendo da inclusão da EAN no processo de ensino e aprendizagem uma das diretrizes do PNAE.

Essa diretriz, alinhada ao conceito de EAN no contexto da realização do DHAA e da garantia da segurança alimentar e nutricional, adotada pelo Brasil no Marco de Referência de Educação Alimentar e Nutricional para as Políticas Públicas (23), foi traduzida para o PNAE na Resolução/CD/FNDE 26, de 17 de junho de 2013 (15), que define EAN como

... o conjunto de ações formativas, de prática contínua e permanente, transdisciplinar, intersetorial e multiprofissional, que objetiva estimular a adoção voluntária de práticas e escolhas alimentares saudáveis que colaborem para a aprendizagem, o estado de saúde do escolar e a qualidade de vida do indivíduo (artigo 13).

A partir de então, a gestão do PNAE passou a adotar e incorporar, também, os princípios do Marco de Educação Alimentar e Nutricional no processo de elaboração de estratégias, instrumentos e propostas de ação de EAN, tendo como referência o Guia alimentar para a população brasileira (25).

## O Programa Nacional do Livro e do Material Didático

A escola é um espaço privilegiado para o desenvolvimento de ações de EAN, considerando, entre outros, o fornecimento de uma alimentação adequada e saudável e o currículo escolar. Nesse sentido, o livro didático se apresenta como uma ferramenta valorosa para a prática de EAN (24).

Recurso pedagógico presente em todas as escolas brasileiras, o livro didático é distribuído pelo governo federal por meio do FNDE. Diferentemente do que ocorre com a alimentação escolar, a autarquia não repassa recursos para as aquisições de livros didáticos, acervos de obras literárias, obras complementares e dicionários (26); em vez disso, adquire e fornece o livro e o material didático às escolas de educação básica pública, de forma universal e gratuita (6).

O PNLD assumiu seu formato atual em meados da década de 1990; entretanto, sua origem remonta a 1929, com a criação do Instituto Nacional do Livro (INL), órgão específico para legislar sobre políticas do livro didático. Contudo, seu funcionamento se iniciou somente em 1934, assumindo um caráter geral de incentivo à leitura (27). Por meio do PNLD, os professores e a escola podem escolher, dentre as opções aprovadas, os livros didáticos que melhor atendam ao projeto político pedagógico e ao contexto social e cultural dos estudantes. Além da relevância do PNLD no que se refere aos aspectos pedagógicos do livro didático, o FNDE, ao adquirir livros para todas as unidades escolares públicas, interfere no setor ligado à produção de livros como agente de controle e consumidor dessa produção, pois os investimentos do PNLD são elevados, tornando o programa um dos maiores do mundo (28).

## PROCESSO DIALÓGICO E INTERSETORIAL NA PROMOÇÃO DE EDUCAÇÃO ALIMENTAR E NUTRICIONAL

Como citado anteriormente, a gestão central do PNAE adotou, por meio da EAN, os princípios do Marco de EAN no processo de elaboração de estratégias de promoção da alimentação adequada e saudável no ambiente escolar. Dentre os princípios, citam-se a intersetorialidade, a educação enquanto processo permanente e gerador de autonomia e a participação ativa e informada dos sujeitos. Dessa forma, espaços intersetoriais de construção coletiva, dialógica e multiprofissional foram criados, partindo da premissa de que o desenvolvimento de ações educativas que auxiliem os estudantes a se tornarem produtores sociais de sua saúde implica no seu empoderamento, gerando autonomia e autocuidado.

**FIGURA 1.**
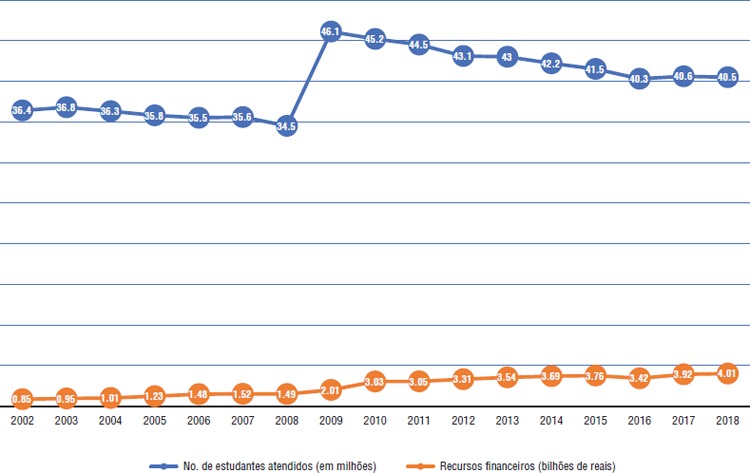
Recursos financeiros repassados pelo Fundo Nacional de Desenvolvimento da Educação ao Programa Nacional de Alimentação Escolar e número de estudantes atendidos, Brasil, 2002 a 2018

Aliado ao compromisso de apoiar ações de EAN em 100 mil escolas de educação básica assumido pelo MEC no Plano Plurianual 2016-2019 (29), o FNDE decidiu investir na convergência do PNLD e do PNAE, de forma ampliada e abrangente, a fim de apresentar aos estudantes e docentes os temas relacionados à segurança alimentar e nutricional. Juntos, os programas movimentam valores impactantes no orçamento do governo federal na área educacional (6, 7), conforme apresentados na tabela 1 e na figura 1.

**TABELA 1.** Resultados do Programa Nacional do Livro e do Material Didático segundo modalidade de atendimento, Brasil, 2015 a 2018

Ano/série escolar^a^	Escolas públicas atendidas	Estudantes beneficiados	Exemplares dos livros didáticos distribuídos	Valores de aquisição e distribuição (R$) dos livros didáticos
2015				
1º ao 5º ano ensino fundamental	47 225	10 764 129	25 454 102	203 899 968,88
6º ao 9º ano ensino fundamental	51 762	10 774 512	27 605 870	227 303 040,19
1ª a 3ª série ensino médio	19 363	7 112 492	87 622 022	898 947 328,29
PNLD Campo 2015	58 180	1 950 211	3 609 379	32 467 996,65
Total	123 947	30 601 344	144 291 373	1 362 618 334,01
2016				
1º ao 5º ano ensino fundamental	39 606	10 150 460	47 409 364	426 790 678,05
6º ao 9º ano ensino fundamental	51 439	10 995 258	28 170 038	275 133 673,10
1ª a 3ª série ensino médio	19 538	7 405 119	35 337 412	371 289 490,61
PNLD Campo 2016	59 097	2 609 633	9 901 805	77 799 184,25
PNLD EJA 2016	25 536	3 352 605	7 770 111	104 482 963,81
Total	121 574	34 513 075	128 588 730	1 255 495 989,82
2017				
1º ao 5º ano ensino fundamental	40 309	9 854 439	31 906 692	317 183 025,76
6º ao 9º ano ensino fundamental	49 702	10 238 539	79 216 538	757 738 473,45
1ª a 3ª série ensino médio	20 228	6 830 011	33 611 125	386 947 980,07
PNLD Campo 2017	56 323	2 493 522	7 617 408	68 278 582,29
PNLD EJA 2017	29 431	2 718 526	4 992 386	83 037 977,27
Total	117 690	32 135 037	157 344 149	1 613 186 038,84
2018				
1º ao 5º ano ensino fundamental	39 465	9 569 765	26 359 755	284 102 621,11
6º ao 9º ano ensino fundamental	46 312	9 818 107	27 615 896	295 860 641,27
1ª a 3ª série ensino médio	19 921	7 085 669	89 381 588	1 036 245 428,10
PNLD Campo 2018	55 619	2 588 165	7 167 788	70 297 229,31
PNLD EJA 2018	28 488	2 075 973	3 374 120	52 125 777,04
Total	117 566	31 137 679	153 899 147	1 738 631 696,82

***Fonte***: FNDE, 2019 (3).

a EJA: Educação de Jovens Adultos; PNLD: Programa Nacional do Livro e do Material Didático. PNLD Campo: livros distribuídos para escolas da área rural.

A partir do ano de 2015, em consonância com a normatização robusta dos alimentos a serem adquiridos pelo PNAE, a qual proíbe a aquisição de bebidas de baixo valor nutricional, restringe a aquisição de alimentos ultraprocessados e obriga o gestor a incluir frutas *in natura* e hortaliças semanalmente, iniciaram-se ações de interação do PNAE com o PNLD, associadas a um processo educativo de promoção da alimentação saudável. O primeiro movimento resultou na revisão dos conteúdos dos editais de convocação para o processo de inscrição e avaliação das obras didáticas para o PNLD, que passaram a incluir, no rol dos critérios eliminatórios comuns, a obrigatoriedade de abordar atividades e propostas temáticas voltadas para a valorização dos cuidados com a alimentação saudável.

Em um segundo momento, as ações se voltaram para a alteração das capas das obras a serem distribuídas aos estudantes. Até 2015, o hino nacional brasileiro esteve presente na quarta capa do livro didático. A partir de 2016, a quarta capa passou a veicular mensagens sobre alimentação saudável, com a proposta da inclusão da EAN nas escolas públicas da educação básica, e o hino nacional passou a ser impresso na terceira capa. Considerando o princípio da intersetorialidade, as capas foram produzidas em parceria com a Coordenação Geral de Alimentação e Nutrição (CGAN) do Ministério da Saúde.

**FIGURA 2.**
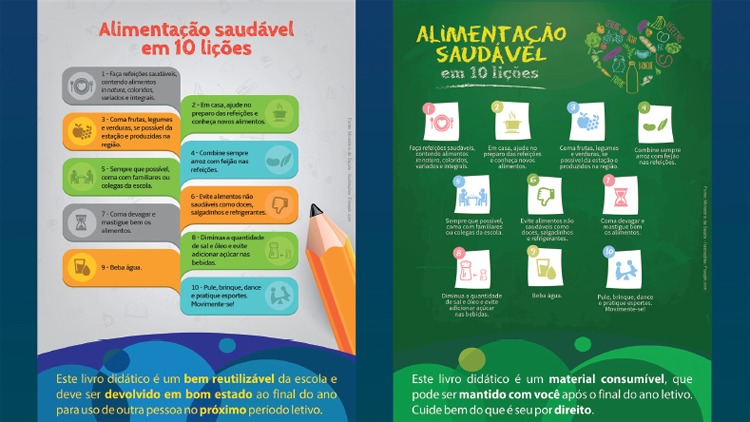
Quartas capas dos livros distribuídos no ensino fundamental pelo Programa Nacional do Livro e do Material Didático no Brasil, 2016 e 2017

**FIGURA 3.**
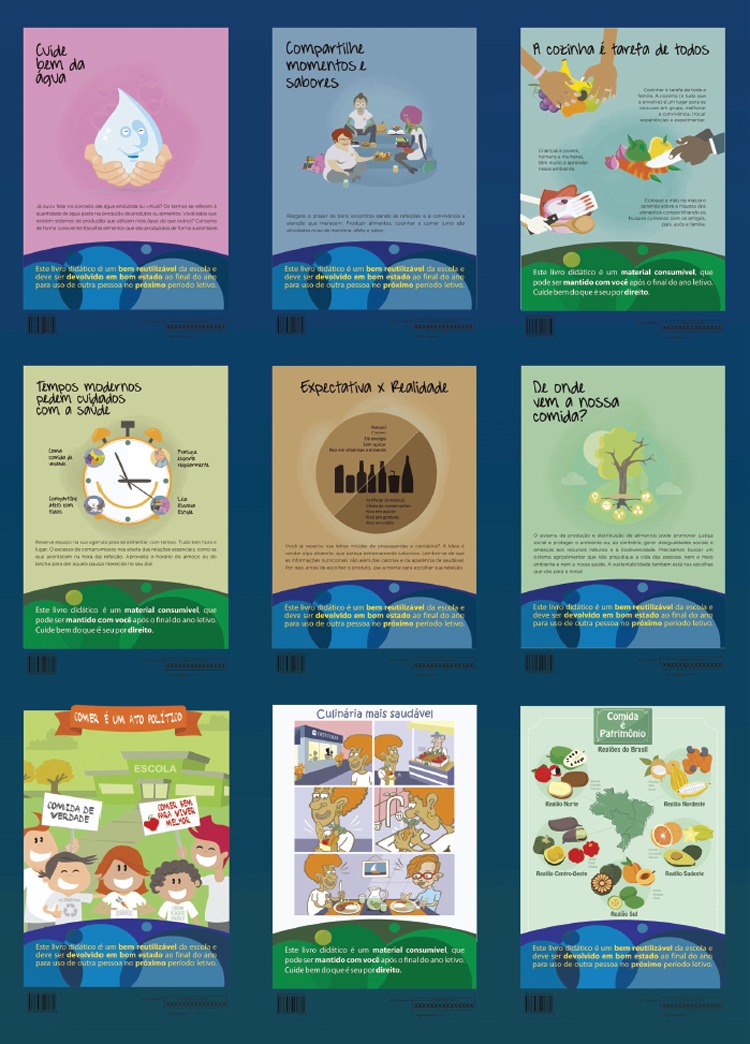
Quartas capas dos livros distribuídos para o ensino médio (1º ao 3º ano) pelo Programa Nacional do Livro e do Material Didático no Brasil, 2018

Em 2017, o FNDE, ainda em parceria com a CGAN, elaborou as capas das obras a serem distribuídas para o ensino médio em 2018, contando com a parceria do Ministério da Cidadania por meio do movimento “Comer pra quê?” (http://mds.gov.br). Os temas utilizados para a construção das capas foram os temas mobilizadores do movimento: Tempos modernos, Comer é um ato político, Todos juntos e misturados, A comida é nossa, De onde vem nossa comida, Come-se propaganda?, Você já comeu água hoje? e Juntin ou rapidin?. Cada quarta capa aborda um tema mobilizador específico, que busca despertar o interesse e a motivação dos professores para o planejamento e a prática de atividades educativas abordando o tema alimentação, nutrição e práticas saudáveis de vida, na perspectiva da segurança alimentar e nutricional (figuras 2, 3 e 4).

Em outra ação intersetorial, o FNDE, dessa vez por meio do Centro Colaborador em Alimentação e Nutrição do Escolar (CECANE), que consiste em descentralização orçamentária do governo federal para universidades federais brasileiras, produziu as quartas capas para os anos finais do ensino fundamental (6º ao 9º ano), distribuídas pelo PNLD 2019, por meio da Universidade Federal do Estado do Rio de Janeiro (UNIRIO). Além das capas, o CECANE UNIRIO também propôs peças de comunicação e materiais de apoio à realização de ações de EAN articuladas ao currículo escolar dos ensinos fundamental e médio.

Para o desenvolvimento dos materiais educativos, o CECANE se baseou em resoluções referentes à alimentação escolar; no Guia Alimentar para a População Brasileira; no Marco de Referência de EAN para as Políticas Públicas; em estudos atualizados sobre a alimentação e nutrição de crianças, adolescentes e jovens brasileiros; e em estudos sobre estratégias de ensino-aprendizagem e de comunicação adequadas a este público no contexto atual. Para a produção das peças de comunicação, foram desenvolvidas estratégias de formação de arcabouço teórico e metodológico e oficinas de criação de materiais educativos (protótipos).

Nesse sentido, os Parâmetros Curriculares Nacionais, a Base Nacional Comum Curricular e o caderno de atividades do Programa Saúde na Escola foram as principais referências básicas. Para o processo de elaboração das capas, utilizou-se uma abordagem metodológica de construção coletiva do conhecimento, por meio das oficinas com a participação de uma rede de parceiros estratégicos (educadores, pedagogos, nutricionistas, profissionais da comunicação, e outros profissionais que trabalham com escolares).

## CONSIDERAÇÕES FINAIS

No Brasil, os avanços do PNAE ao longo das seis décadas foram resultados de decisões políticas amparadas pela sociedade civil que alçaram o programa ao patamar de uma política de Estado, de consolidação do direito à alimentação adequada e saudável e de concepções estruturantes na perspectiva da segurança alimentar e nutricional. Do mesmo modo, o PNLD é uma ferramenta à disposição do gestor para promover hábitos alimentares saudáveis nas escolas, com abrangência que ultrapassa as fronteiras das unidades escolares, atingindo as famílias dos estudantes.

Tendo em vista o valor da EAN como instrumento da promoção da alimentação adequada e saudável, a ampla presença dos livros didáticos nas escolas e a posição privilegiada que os mesmos possuem para promover e direcionar práticas pedagógicas dentro do contexto escolar, verifica-se que a construção de um espaço dialógico e intersetorial entre diversos atores governamentais é um caminho possível de promover a saúde dos estudantes. Com a participação de equipes de técnicos comprometidos e de órgãos, entidades e docentes que militam na educação e na saúde pública, é possível construir, nos programas educacionais, ações governamentais potencializadoras, de abrangência nacional e convergentes na promoção da alimentação adequada e saudável.

Finalmente, sugere-se a realização de estudos que avaliem a percepção dos docentes e discentes quanto às alterações realizadas nas capas dos livros do PNLD. Além disso, deve-se avaliar, também, se as mensagens contidas nas quartas capas estão promovendo discussões e aquisição de conhecimentos que possam gerar habilidades para uma leitura crítica sobre alimentação, sendo capazes de se consolidarem como uma ferramenta relevante no processo educativo dos temas de alimentação e nutrição.

## Contribuição dos autores.

SFFC elaborou a versão inicial e aprovou a versão final. MBM e VMGS deram contribuições substanciais ao manuscrito e aprovaram a versão final. KSS contribuiu com a revisão e aprovou a versão final.

**FIGURA 4.**
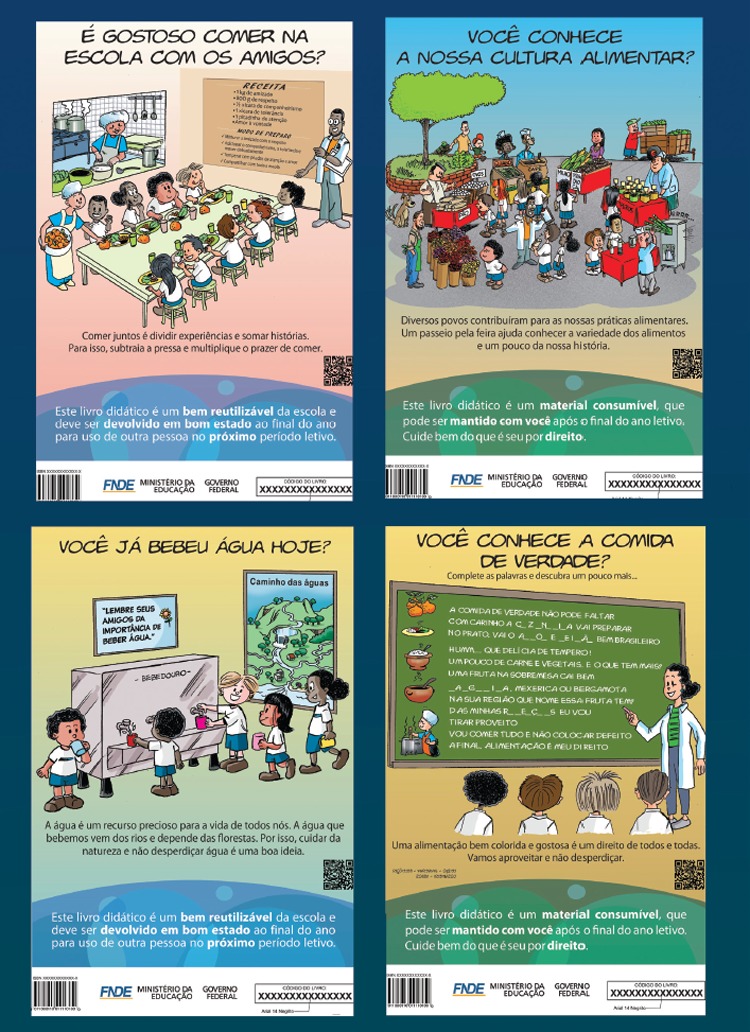
Quartas capas dos livros distribuídos aos anos iniciais do ensino fundamental pelo Programa Nacional do Livro e do Material Didático no Brasil, 2019

## Conflito de interesses.

Nada declarado pelos autores.

## Declaração.

As opiniões expressas no manuscrito são de responsabilidade exclusiva dos autores e não refletem necessariamente a opinião ou política da RPSP/PAJPH ou da Organização Pan-Americana da Saúde (OPAS).

## References

[1] 1. Brasil, Constituição 1988. Constituição da República Federativa do Brasil. Brasília: Senado Federal; 1988. Disponível em http://www.planalto.gov.br/ccivil_03/constituicao/constituicao.htm Acessado em outubro de 2019.

[2] 2. United Nations, General Assembly. Resolution adopted by the General Assembly on 1 April 2016. 70/259. United Nations Decade of Action on Nutrition (2016–2025). Disponível em: https://undocs.org/pdf?symbol=en/A/RES/70/259 Acessado em setembro de 2019.

[3] 3. Câmara Interministerial de Segurança Alimentar e Nutricional. Compromissos do Brasil para a Década de Ação das Nações Unidas para a Nutrição (2016-2025). Brasília: Câmara Interministerial de Segurança Alimentar e Nutricional; 2017.

[4] 4. Câmara Interministerial de Segurança Alimentar e Nutricional. II Plano Nacional de Segurança Alimentar e Nutricional - 2016/2019. Brasília: Ministério do Desenvolvimento Social e Agrário; 2017. Disponível em: http://www.mds.gov.br/webarquivos/arquivo/seguranca_alimentar/caisan/Publicacao/Caisan_Nacional/PLANSAN%202016-2019_revisado_completo.pdf Acessado em setembro de 2019.

[5] 5. Câmara Interministerial de Segurança Alimentar e Nutricional. Compromissos do Brasil para a Década de Ação das Nações Unidas para a Nutrição (2016-2025). Brasília: Ministério do Desenvolvimento Social; 2017. Disponível em: http://mds.gov.br/caisan-mds/publicacoes/decada_versao_portugues.pdf Acessado em setembro de 2019.

[6] 6. Brasil, Ministério da Educação. Fundo Nacional de Desenvolvimento da Educação. Programas do Livro. Disponível em: http://www.fnde.gov.br/index.php/programas/programas-do-livro/pnld/dados-estatisticos Acessado em 25 de novembro de 2018.

[7] 7. Brasil, Ministério da Educação. Fundo Nacional de Desenvolvimento da Educação. Dados abertos: PNAE. Disponível em: http://www.fnde.gov.br/dadosabertos/dataset/repasses-financeiros-do-pnae Acessado em 8 de novembro de 2018.

[8] 8. ActionAid. Contribuições do PAA e do PNAE à construção da segurança alimentar e nutricional. A experiência brasileira. Rio de Janeiro: ActionAid; 2016. Disponível em: http://actionaid.org.br/wp-content/files_mf/1493417778relatorioaexperienciabrasileira.pdf Acessado em 8 de agosto de 2019.

[9] 9. Brasil. Lei 11 947/2009. Diário Oficial da União, 16 de junho de 2009. Disponível em: http://www.planalto.gov.br/ccivil_03/_Ato2007-2010/2009/Lei/L11947.htm Acessado em agosto de 2019.

[10] 10. Triches RM, Schneider S. Alimentação escolar e agricultura familiar: reconectando o consumo à produção. Saude Soc. 2010;19(4):933-45.

[11] 11. Chaim N, Belik W. São Bernardo do Campo: atuação pioneira em favor da agricultura familiar. Em: Corá MAJ, Belik W, eds. Projeto Nutre SP: análise da inclusão da agricultura familiar na alimentação escolar no estado de São Paulo. Brasília: Ministério do Desenvolvimento Agrário; 2012. Pp. 77-88.

[12] 12. Schwartzman F. Vinculação do Programa Nacional de Alimentação Escolar (PNAE) com a agricultura familiar: caracterização da venda direta e das mudanças para os agricultores familiares em municípios do estado de São Paulo [tese de doutorado]. São Paulo: Faculdade de Saúde Pública, Universidade de São Paulo; 2015. Disponível em: https://teses.usp.br/teses/disponiveis/6/6138/tde-26052015-093714/pt-br.php Acessado em agosto de 2019.

[13] 13. Teo CRPA, Motter AF, Barbosa LP, Dacroce M, Pagliarini G. Articulação entre agricultura familiar e alimentação escolar em municípios de pequeno porte. Campo Territorio. 2016;11(24):175-99.

[14] 14. Schwartzman F, Mora CAR, Bogus CM, Villar BS. Background and elements of the linkage between the Brazilian school feeding program and family farming. Cad Saude Publica. 2017;33(12):e00099816. 10.1590/0102-311X00099816 29267684

[15] 15. Brasil, Fundo Nacional de Desenvolvimento da Educação. Resolução CD/FNDE/2013. Disponível em: https://www.fnde.gov.br/index.php/acesso-a-informacao/institucional/legislacao/item/4620-resolu%C3%A7%C3%A3o-cd-fnde-n%C2%BA-26,-de-17-de-junho-de-2013 Acessado em 30 de dezembro de 2018.

[16] 16. Gonçalves EVB, Cunha DT, Stedefeldt E, Rosso VV. Family farming products on menus in school feeding: a partnership for promoting healthy eating. Cienc Rural. 2015;45(12):2267-73.

[17] 17. Bezerra JAB. Educação Alimentar e Nutricional: articulação de saberes. Fortaleza: Edições UFC; 2018.

[18] 18. Brasil. Lei 11 346/2006. Diário Oficial da União, 18 de setembro de 2006. Disponível em: http://www4.planalto.gov.br/consea/conferencia/documentos/lei-de-seguranca-alimentar-e-nutricional Acessado em setembro de 2019.

[19] 19. Brasil. Decreto 7 272/2010.Diário Oficial da União, 26 de Agosto de 2010. Disponível em: http://www.planalto.gov.br/ccivil_03/_Ato2007-2010/2010/Decreto/D7272.htm Acessado em setembro de 2019.

[20] 20. Custodio MB, Yuba TY, Cyrillo DC. Política de segurança alimentar e nutricional no Brasil: uma análise da alocação de recursos. Rev Panam Salud Publica. 2013;33(2):144-50. 10.1590/s1020-49892013000200010 23525345

[21] 21. Amorim ALB, Rosso VV, Bandoni DH. Acquisition of family farm foods for school meals: analysis of public procurements within rural family farming published by the cities of São Paulo state. Rev Nutr. 2016;29(2):297-306.

[22] 22. Rodrigues R, Siqueira HM, Biancardi CCS, Andrade MAN, Valente LM, Paula LB. A aquisição de alimentos da agricultura familiar pelo PNAE no município de Alegre - ES. Demetra. 2017;12(1);91-112.

[23] 23. Brasil, Ministério do Desenvolvimento Social e Combate à Fome, Secretaria Nacional de Segurança Alimentar e Nutricional. Marco de referência de educação alimentar e nutricional para as políticas públicas. Brasília: Secretaria Nacional de Segurança Alimentar e Nutricional; 2012. Disponível em: http://www.mds.gov.br/webarquivos/arquivo/seguranca_alimentar/caisan/Publicacao/Educacao_Alimentar_Nutricional/1_marcoEAN.pdf Acessado em agosto de 2019.

[24] 24. Greenwood SA, Fonseca AB. Espaços e caminhos da educação alimentar e nutricional no livro didático. Cienc Educ. 2016;22(1):201-18.

[25] 25. Brasil, Ministério da Saúde. Guia Alimentar para a População Brasileira. 2a ed. Brasília: Ministério da Saúde; 2014. Disponível em: http://bvsms.saude.gov.br/bvs/publicacoes/guia_alimentar_populacao_brasileira_2ed.pdf Acessado em outubro de 2019.

[26] 26. Brasil, Ministério da Educação. Portal do MEC. Disponível em: http://portal.mec.gov.br/busca-geral/318-programas-e-acoes-1921564125/pnld-439702797/12391-pnld Acessado em 2 de agosto de 2019.

[27] 27. Di Giorgi CAG, Militão SCN, Militão NA, Perboni F, Ramos RC, Lima VMM, et al. Uma proposta de aperfeiçoamento do PNLD como política pública: o livro didático como capital cultural do aluno/família. Ensaio: Aval Pol Publ Educ. 2014;22(85):1027-56.

[28] 28. Mantovani KP. O Programa Nacional do Livro Didático - PNLD: impactos na qualidade do ensino público [dissertação]. São Paulo: Faculdade de Filosofia, Letras e Ciências Humanas da Universidade de São Paulo; 2009. Disponível em: https://teses.usp.br/teses/disponiveis/8/8136/tde-24112009-152212/pt-br.php Acessado em agosto de 2019.

[29] 29. Brasil, Ministério do Planejamento, Orçamento e Gestão. Plano Plurianual 2016-2019: desenvolvimento, produtividade e inclusão social – mensagem Presidencial. Disponível em: http://www.planejamento.gov.br/secretarias/upload/arquivo/spi-1/ppa-2016-2019/ppa-2016-2019-ascom-3-1.pdf Acessado em 19 de novembro de 2018.

